# Increased Urge to Gamble Following Near-Miss Outcomes May Drive Purchasing Behaviour in Scratch Card Gambling

**DOI:** 10.1007/s10899-016-9662-2

**Published:** 2016-12-24

**Authors:** Madison Stange, Candice Graydon, Mike J. Dixon

**Affiliations:** Department of Psychology, University of Waterloo, Waterloo, ON N2L 3G1 Canada

**Keywords:** Scratch cards, Gambling, Near-misses, Motivation, Purchasing behaviour

## Abstract

Previous research into scratch card gambling has highlighted the effects of these games on players’ arousal and affective states. Specifically, near-miss outcomes in scratch cards (uncovering 2 of 3 needed jackpot symbols) have been associated with high levels of physiological and subjective arousal and negative emotional evaluations, including increased frustration. We sought to extend this research by examining whether near-misses prompted increases in gambling urge, and the subsequent purchasing of additional scratch cards. Participants played two scratch cards with varying outcomes with half of the sample experiencing a near-miss for the jackpot prize, and the other half experiencing a regular loss. Players rated their urge to continue gambling after each game outcome, and following the initial playing phase, were then able to use their winnings to purchase additional cards. Our results indicated that near-misses increased the urge to gamble significantly more than regular losses, and urge to gamble in the near-miss group was significantly correlated with purchasing at least one additional card. Although some players in the loss group purchased another card, there was no correlation between urge to gamble and purchasing in this group. Additionally, participants in the near-miss group who purchased additional cards reported higher levels of urge than those who did not purchase more cards. This was not true for the loss group: participants who experienced solely losing outcomes reported similar levels of urge regardless of whether or not they purchased more scratch cards. Despite near-misses’ objective status as monetary losses, the increased urge that follows near-miss outcomes may translate into further scratch card gambling for a subset of individuals .

## Introduction

Scratch cards (also referred to as “instant tickets” or “instant win” games) are a ubiquitous form of gambling in our society. Many different types of scratch-card games exist, but in general, the goal of these games is to uncover matching symbols by removing an opaque film covering. Players typically remove this covering by “scratching” it off with the aid of a small coin. Depending on what is matched or uncovered, a certain prize may be attained. In Canada, scratch cards range in price from $1.00 to $30.00, with the majority of cards being in the $3.00 to $10.00 range (*M* = $5.81; calculated from OLG 2016). The payback percentages of scratch-card games in Ontario range from 59.97 to 70.39% (*M* = 65.73%; calculated from OLG 2016).

Researchers have long focused on the use of these products by youth, with lottery products being a highly sought-after type of gambling for this demographic (Griffiths 2000; Felsher et al. 2004; Donati et al. 2013; Wood and Griffiths 1998). A recently published study of Canadian youth aged 13–19 found that scratch cards were the most common type of regulated gambling behaviour engaged in by these teenagers, with 13.8% of the sample endorsing participation (Elton-Marshall et al. 2016). In light of these findings, researchers have begun to examine in more detail the types of individuals who play these games and the experiences associated with this form of gambling.

In a large Canadian survey study (Short et al. 2015) the amount of scratch-card gambling that participants engaged in was negatively correlated with level of education; no other demographic variables (e.g., age, sex, marital status) were meaningfully correlated with frequency of scratch-card play. Another study found that Ontario baby boomers who played scratch cards reported participating in more forms of gambling than those in the cohort who did not report playing these games (Papoff and Norris 2009). These authors also found that at-risk/problem gambling prevalence was significantly higher among respondents who purchased scratch cards compared to those who did not. Similarly, a large, 5-year longitudinal study of gambling behaviour in Canada found that instant-win-ticket gambling (which includes scratch cards) was predictive of problem gambling over time (Williams et al. 2015). Although population estimates for pathological scratch card gambling are low (DeFuentes-Merillas et al. 2003), case study reports of pathological scratch card gamblers do exist (Raposo-Lima et al. 2015), demonstrating that for a small portion of gamblers, these games may be associated with problematic use. Although studies examining player characteristics are informative, examining specific structural aspects of the games themselves may also help to elucidate how these games affect the people who play them. Such knowledge, in turn may provide insight into why these games are so popular with the general population.

While typically seen as an innocuous type of gambling, scratch cards nevertheless bear many similarities to more addictive forms of gambling. Specifically, these games share many structural characteristics with slot machines. These include intermittent payout intervals, rapid event frequency, the opportunity for continual play, and near-miss outcomes (Griffiths 1995a, b, 1997; Wood and Griffiths 1998). Multiple authors have commented on these resemblances, referring to these games as slot machines in a paper form (Griffiths 1995b, 1997; Ariyabuddhiphongs 2011), and consequently a potentially “hard” (as opposed to a “softer”, more innocuous) form of gambling (Griffiths 2002). Of the many surface similarities between slot machines and scratch cards, arguably the most striking are near-misses. Since near-misses have been most rigorously investigated in the context of slot machine play, we turn first to this literature to inform our predictions.

Slot machines have been associated with high levels of problem gambling (Dowling et al. 2005) for quite some time, and across a wide range of countries (Fisher and Griffiths 1995). Tellingly, the Ontario Problem Gambling Helpline receives more calls identifying slot machine gambling as a concern compared to any other gambling type (Counter and Davey 2006). As previously mentioned, near-misses are a ubiquitous feature of many slot machine games. Reid (1986) defined a near-miss as an outcome that comes close to a win but falls short. A classic near-miss in a slot machine game consists of two of the required jackpot symbols landing on the payline, with the third landing just below or above. In a scratch card game, a near-miss consists of players getting two of the three symbols required to win a jackpot prize, but missing the third. These outcomes create the appearance of coming close to a jackpot, but are nonetheless a losing outcome, in that there is no monetary gain for the player. The effects of near-miss outcomes on gamblers have been particularly well documented in slot machine research, with a wealth of studies describing the effects of these outcomes in human participants (Clark et al. 2013; Dixon et al. 2013; Habib and Dixon 2010), rats (Winstanley et al. 2011) and pigeons (Scarf et al. 2011)

Many studies of human gamblers report that slot machine near-misses increase players’ physiological arousal, as measured by skin conductance (or electrodermal activity) and heart rate (Clark et al. 2012, 2013; Dixon et al. 2011, 2013). Increased arousal for near-miss outcomes may be problematic, as heightened physiological arousal has been identified as a key reinforcer of gambling behaviour (Brown 1986). As such, if arousal is reinforcing, and near-misses trigger an increase in arousal, then it could be that players are being reinforced for losing. Heightened arousal that accompanies a lack of goal attainment can result in a paradoxically frustrating, yet highly motivating subjective experience. In line with this notion, some authors have postulated that heightened arousal indicates enhanced motivation (Bradley and Lang 2007).

Consistent with the notion that near-misses increase motivation, near-miss outcomes in slot machines have been shown to prolong the amount of time spent gambling (Côté et al. 2003; Kassinove and Schare 2001). Neuroimaging studies allow insight into the mechanisms behind these behavioural effects. Near-miss outcomes in a simulated slot machine task have been found to activate the ventral striatum, an area associated with reward processing (Clark et al. 2009), despite their objective status as a losing outcome. On this same task, participants rated near-miss outcomes as unpleasant, yet still motivating when they had personal control over their wager. These results highlight the paradoxically motivating yet aversive nature of near-miss outcomes, and the behavioural consequences that they have for the gambler (i.e. increased money and time spent gambling). Indeed, research on approach motivation suggests that motivated behaviour can occur in response to negative stimuli (Harmon-Jones et al. 2013). Thus near-misses may activate the “wanting” as opposed to the hedonic “liking” facet of the reward system (Berridge 2007).

Research regarding near-misses in slot machines has prompted us to investigate the effects of near-miss outcomes in scratch cards. We found that players showed heightened physiological arousal as they uncovered the symbols that led to small wins and near-miss outcomes. We showed such effects using both skin conductance (Stange et al. 2016b) and heart rate changes (Stange et al. 2016a). Additionally, in these studies near-miss outcomes were consistently rated as the most frustrating and emotionally negative outcome, whereas wins were rated as the most positive and least frustrating. Importantly, scratch card near-misses also appeared to increase the urge to continue gambling. When student scratch-card gamblers were polled immediately following each outcome, urge to continue gambling was as elevated following near-miss outcomes as it was for small wins of $5.00 (Stange et al. 2016a). These results suggest that scratch card near-misses, even though they are monetary losses, may be capable of encouraging further gambling behaviour much like their slot machine equivalents.

Although we have shown increases in arousal, frustration, negative affect, and subjective urge following scratch card near-misses, it remains unknown whether or not experiencing these outcomes would actually prolong gambling behaviour, as in slot machines. In this study our two overarching goals were to: (1) replicate our previous finding that near-miss outcomes trigger increases in the urge to gamble, and (2) assess whether near-misses and their associated heightened urge would prompt participants to actually purchase more scratch cards. We had participants play two custom-made scratch cards. On the first card (Card 1), all participants experienced a loss, a small win and another loss. On the second card (Card 2), one group of participants experienced three consecutive losing games, while the other group experienced two losses, followed by a near miss. Participants were asked to give ratings of their urge to gamble after each outcome. Following game play, participants were given an opportunity to use their winnings (from Card 1) to purchase additional cards. We predicted that participants would experience increases in the urge to gamble following both winning and near-miss outcomes (a replication of our previous findings). We also predicted that participants who experienced a near-miss outcome would be more likely than participants who experienced only losses to use their winnings to purchase additional cards. Finally, we predicted that this purchasing behaviour would be attributable to increases in the urge to continue gambling following the near-miss outcome, as compared to regular losing outcomes.

## Method

### Participants

Participants gave informed written consent before the study began, and all procedures were approved by the University of Waterloo’s Office of Research Ethics. Sixty-five undergraduate students were recruited from the University of Waterloo’s Research Experience Group in exchange for course credit. All participants were prescreened to ensure that they were at least 18 years of age (the legal age to purchase scratch cards in Ontario), had experience playing scratch cards, and were not currently in or seeking treatment for problem gambling. The average age of the participants was 19.97 years (*SD* = 1.57), and the sample was predominantly female (51 females, 14 males). One participant was excluded from all analyses due to a procedural error, and six were excluded due to incomplete data (see “Analytical Strategy” section).

### Instruments and Materials

#### Problem Gambling Severity Index

The Problem Gambling Severity Index (PGSI) is a subscale of the Canadian Problem Gambling Index (CPGI), a well-validated screen for gambling problems and overall problem gambling severity in the general population (Ferris and Wynne 2001). This measure was used to characterize our sample; no specific hypotheses concerning problem gambling status were made.

#### Gambling Related Cognitions Scale

The Gambling Related Cognitions Scale (GRCS; Raylu and Oei 2004) was administered for purposes peripheral to this study and will not be discussed further.

#### Measure of Gambling Urge

To assess participants’ urge to gamble, we used the following item: “How would you rate your desire to gamble on a scale from 0 (no desire to gamble) to 100 (overwhelming desire to gamble)?” (Young et al. 2008). Participants responded by moving a cursor along a linear sliding scale (ranging from 0 to 100) to the location that best reflected their urge to gamble.

#### Scratch Cards

**Fig. 1:**
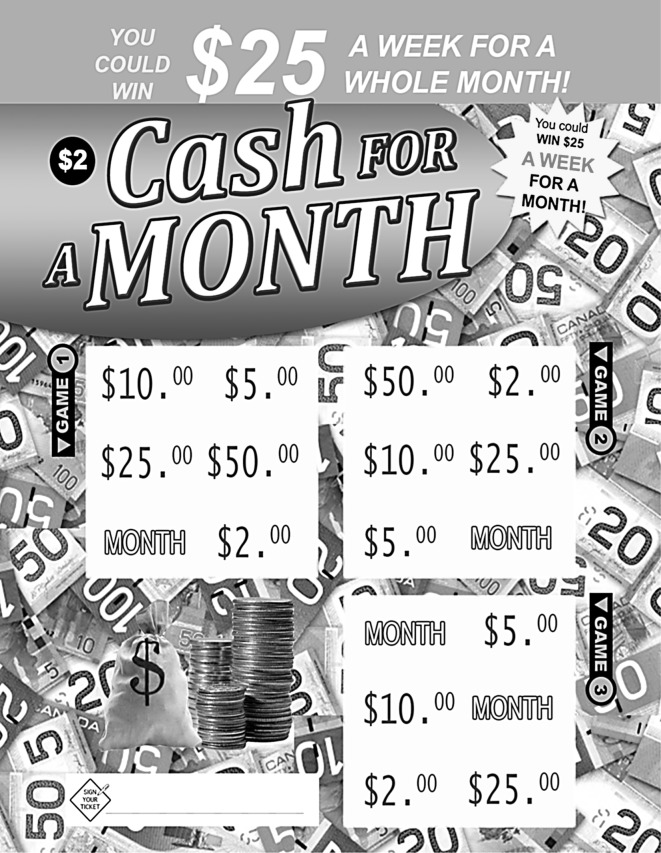
“Cash for a Month” scratch card. The custom made scratch cards employed in this study were designed to mimic a popular scratch card available in Ontario. This card contains two losses (games 1 and 2) and a near-miss for the top prize (game 3)

The custom made scratch cards were modeled after Cash for Life, a scratch card game currently available in Ontario. In Cash for Life, the player is presented with game-play boxes containing symbols denoting various monetary amounts. To win a prize, a player must uncover three matching symbols within one game. The player then wins the amount specified by the symbol (i.e. three matching $5.00 symbols would mean a win of $5.00). Our game utilized a similar game structure and design in that three matching symbols were needed to win a prize. The cards in this study (described in detail below) were similar in design to those used in previous studies (Fig. 1; see also Stange et al. 2016a, b).

### Procedure

Participants were brought into the laboratory, where they signed an informed consent letter. Participants then completed the PGSI (Ferris and Wynne 2001) and demographic items on a laptop computer. Following this, participants were told that the game they would be playing was called “Cash for a Month”, and that it was similar to existing scratch card games available at Ontario retailers. Using an enlarged example of one of the cards, the experimenter showed participants that each scratch card contained three games, and within each game, there were six symbols (Fig. 1). The experimenter explained that the goal of the scratch card game was to find three matching symbols within any one of the games on the card; if participants found three matching symbols, they won the corresponding prize. Participants were instructed to uncover the symbols one game at a time, and to scratch each game from left to right, and top to bottom. Participants were told to rate their desire to continue gambling after each game (three ratings per card) using a tablet computer that was provided (Lenovo Ideatab, model A1000). The experimenter also explained that to win the top prize of “Cash for a Month” (corresponding to $25.00 a week for 4 weeks, $100.00 total) they would need to uncover three “MONTH” symbols within one game (analogous to the “LIFE” symbol in Cash for Life). Participants were also told that they would pick a scratch card to play from a tray of approximately 100 scratch cards, and that one of the cards in the tray was the top prize winning card. They were reminded that the odds of winning were approximately 1 in 100 and then told that the top prize had been won in past studies. Importantly, participants were told that the first two cards that they would be playing were free, but that if they won anything on those two cards, they would be able to use their winnings to purchase additional cards later on in the study. Participants were asked if they had any questions about the game structure or rules before continuing.

The experimenter then had the participant choose the scratch cards that they would play during the experiment. Participants chose their cards from a display case similar to those found in Ontario lottery retailers and identical to what has been used in previous studies (Stange et al. 2016a, b). The scratch cards were arranged in two trays to facilitate our between-subjects manipulation. In the first tray of cards, all cards contained games with a loss, a small win of $5.00, and another loss. The single top prize card was also included in this tray. The card that participants chose from the second tray determined the condition to which the participant was randomly assigned (half loss cards and half near-miss cards). Participants in the loss group chose a card in which all three games were regular losses. Those in the near-miss group chose a card that contained a loss, a second loss, and then a near-miss (two of the three symbols needed to win the jackpot prize). After choosing their cards, the experimenter placed the scratch card in a secure scratching platform (see Stange et al. 2016b for a more detailed description). Participants played the three games on that card, filled out their urge ratings following each game, and repeated this process for their second card.

Once they had completed scratching both cards, the experimenter gave the participant their winnings ($5.00) and told them they could purchase additional scratch cards to play if they wished. The experimenter explained that each card cost $2.00, and would be chosen from another display case, but the overall odds of winning the top prize remained unchanged. If participants chose to play another card, the experimenter kept $2.00 of the participant’s overall winnings (leaving the participant with $3.00), and let the participant choose another card. Participants then completed the scratch card games and corresponding urge ratings in a similar manner as the first two cards. Any additional cards that participants purchased contained only regular losses comprised of symbol arrangements that participants had not encountered on previous cards. Participants who played a third card were given the option to purchase a fourth card (a cost of $2.00, leaving the participant with $1.00). In sum, if participants chose to not purchase, they left with $5.00, purchasing one additional card meant an overall gain of $3.00, and purchasing two cards left the participant with $1.00. No participants in the current sample won the top prize of “Cash for a Month”.

Following the entire game-play portion of the study, participants completed the GRCS. After completing the survey, participants were given their winnings, a feedback letter, and responsible gambling resources.

## Results

### Sample Characteristics

#### PGSI

Scores on the PGSI indicated that 35 participants were non-problem gamblers (score of 0), 27 were low-risk (score of 1–4), 1 was moderate risk (score of 5–7), and 1 participant was a problem gambler (score above 8; Currie et al. 2013). PGSI status was not analyzed further, primarily since no specific predictions were made about the influence of problem gambling status on our dependent variables, but also because of low numbers of problematic gamblers.

### Purchasing Behaviour

Considering all participants, only 31.3% (n = 20) of the total sample of participants (N = 64) elected to purchase at least one additional scratch card with their winnings. In the loss condition, 25.8% (n = 8) of participants purchased at least one additional card. In the near-miss condition, 36.4% (n = 12) of participants purchased at least one additional card. A Chi-square test of independence revealed that these frequencies were not significantly different, *X* ^2^ (1, *N* = 64) = .829, *p* = .362.

### Urge to Continue Gambling

#### Analytical Strategy

Of the 65 participants recruited, 6 participants were excluded from any data analyses involving urge to continue gambling ratings due to incomplete or missing urge evaluations. Mean ratings of urge to continue gambling were calculated following each outcome, and compared across groups (loss vs. near-miss). Given the nature of the design (Card 1 contained a loss, a small win, and a loss; Card 2 contained two losses with the third game dependent on condition), we analyzed the cards separately. For each card we conducted a mixed analysis of variance (ANOVA) with game as the repeated factor, and group as the between-subjects factor. In the case of tests where sphericity assumptions were violated, corrected degrees and freedom and F values are reported. Post-hoc comparisons were conducted using *t* tests, and were evaluated at α/m (Bonferroni correction) to control for familywise error rate.

#### Card 1

**Fig. 2:**
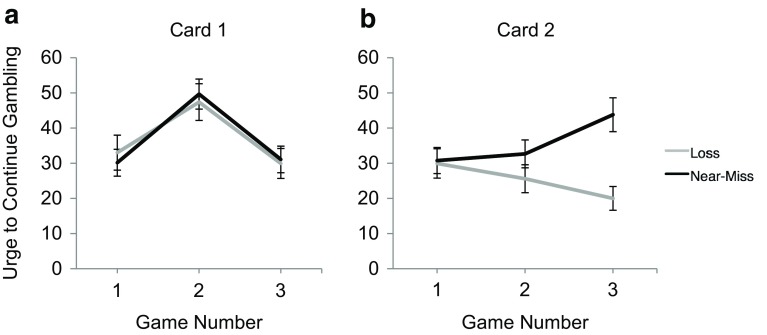
**a** Card 1 urge ratings. Mean urge to continue gambling ratings for participants in the loss and near-miss conditions. Outcomes 1 and 3 were losses, outcome 2 was a small win of $5.00. **b** Card 2 urge ratings. Mean urge to continue gambling ratings for participants in the loss and near-miss conditions. Outcomes 1 and 2 were losses, outcome 3 was a loss for those in the loss condition, but a near-miss for the top prize (Cash for a Month) for those in the near-miss condition. *Error bars* are ±1 SEM

For Card 1 (loss, small win, loss), this analysis indicated a significant main effect of game, *F*(2, 112) = 35.00, *p* < .001, η_p_^2^ = .385. Collapsing across group, post hoc analyses (evaluated at α/2 = .025) indicated that the win triggered higher urge ratings than either the loss preceding it *t*(57) = 7.65, *p* < .001, or following it, *t*(57) = 6.65, *p* < .001. Importantly, the main effect of group (loss, near-miss) was not significant, *F*(1, 56) = .001, *p* = .974. Therefore, there were no pre-existing differences in urge to continue gambling between the groups. The mean urge ratings for Card 1 are shown in Fig. 2a.

#### Card 2

For Card 2, there was no main effect of game, *F*(1.78, 99.85) = 1.04, *p* = .35, η_p_^2^ = .018. There was a main effect of group, *F*(1, 56) = 4.07, *p* = .049, η_p_^2^ = .068. The interpretation of these main effects were qualified by a significant interaction between game number and group, *F*(1.78, 99.85) = 18.96, *p* < .001, η_p_^2^ = .253. This interaction is depicted Fig. 2b. Post hoc *t* tests (evaluated at α/3 = .017) indicated there were no significant differences between the groups for the first loss, *t*(56) = .15, *p* = .88, or the second loss, *t*(56) = 1.26, *p* = .21 but urge ratings at game 3 were significantly higher for those exposed to the near-miss than those exposed to the loss, *t*(56) = 4.04, *p* < .001.

### Relationship Between Urge and Purchase Status

To assess whether different scratch-card outcomes in the very last game on Card 2 (loss or near-miss) fostered differences in post-game urge and subsequent scratch card purchasing behaviour, we conducted point-biserial correlations separately for each group (loss, near-miss), correlating post-outcome urge with purchasing behaviour (non-purchasers coded as 0, purchasers as 1). For the near-miss group, urge ratings immediately following the near-miss were significantly positively correlated with purchasing status, *r*_pb_ = .49, *n* = 29, *p* = .007. For the loss group, however, urge ratings following the loss showed no relationship with purchasing status, *r*_pb_ = -.018, *n* = 29, *p* = .926. Using Fisher’s r-to-z transformations, these correlations were significantly different from each other, Z = 1.99, *p* = .046.

As a supplementary means of assessing whether the near-miss-induced elevations in urge actually triggered purchasing behaviour, we compared the urge levels of purchasers to non-purchasers. We reasoned that if near-misses triggered increases in urge for at least some participants, that those participants should be the ones who would be most likely to purchase additional cards. If so, then purchasers should show higher urge levels than non-purchasers. A between-subjects ANOVA, with group and purchase status as the between-subjects variables indicated a significant interaction between group and purchase status, *F*(1, 54) = 4.90, *p* = .031, η_p_^2^ = .083. Follow-up *t* tests (evaluated at α/2 = .025) indicated that there were no significant differences in urge between participants who did and did not purchase additional cards in the loss group, *t*(27) = .09, *p* = .926. However, for participants in the near-miss condition, purchasers showed significantly higher urge ratings than those who did not purchase additional cards, *t*(27) = 2.92, *p* = .007. Table 1 displays the means and standard deviations of urge to continue gambling for participants in each condition.

**Table 1:** Mean urge ratings at purchase point (Card 2, game 3) by condition and purchase status

Purchase status	Condition	
	Loss	Near-miss
Non-purchasers	20.18 (20.41)	34.74 (25.15)
Purchasers	19.43 (9.64)	61.00 (17.94)*

* Statistically significant differences between purchase status at *p* < .01

## Discussion

We ran an experiment to determine whether near-misses would trigger increases in gambling urge, and whether this increased desire to continue gambling would translate into participants using their winnings to purchase additional scratch cards. Near-misses dramatically increased the urge to gamble—a finding that replicates our previous study on scratch card players (Stange et al. 2016a). Figure 2a shows that the random assignment of players into the two groups was effective—there were no differences between the urge ratings of the groups prior to the key manipulation (the introduction of the near-miss for one of the groups). Figure 2b shows that the groups continued to show similar urge trajectories for the two losses on Card 2. The groups only diverged following the third game when the key manipulation was delivered (a near-miss for half of the participants, and another loss for the other half of the participants). Those who experienced a loss in their third game showed a decline in their urge to gamble, whereas those who experienced a near miss showed a clear spike in gambling urge. In sum, the finding that scratch card near-misses trigger increases in the urge to gamble is a robust one that replicates across studies using different procedures (e.g., the within-subjects design in Stange et al. 2016a, and the between-subjects design employed in the present study).

The effects of near-misses on urge in various gambling forms is at first glance counterintuitive, as they are clearly a monetary loss, yet still enhance the motivation to play. Classic interpretations of near-miss effects derived from investigations of slot-machine play focus on the arousing yet frustrating properties of these outcomes. Slot machine near-misses are consistently reported as being unpleasant outcomes (Clark et al. 2009) that increase physiological arousal (Dixon et al. 2011) and frustration (Dixon et al. 2013). Despite such negative affect, they have been found to prolong slot-machine play (Côté et al. 2003; Kassinove and Schare 2001). Thus a theorized chain of events is as follows: when a player experiences a near-miss, frustration ensues due to having *just* missed the jackpot prize. This is coupled with an increase in physiological arousal and negative subjective evaluations. Due to this state of heightened frustration and physiological arousal, players are eager to move on to their next available game as quickly as possible leading to increases in the urge to continue gambling. This urge then translates, for at least some players, into prolonged or additional gambling behaviour.

In this and previous studies we provide converging evidence for this chain of events in scratch-card play. Near-miss outcomes in scratch cards are associated with increased physiological and subjective arousal, and heightened subjective negative emotion and frustration (Stange et al. 2016a, b). Yet, regardless of their objective monetary status, near-misses have distinct motivational consequences for the player. In the current study they served to increase the urge to gamble compared to those who were exposed to a standard losing outcome.

Our second prediction was that the spikes in urge caused by the near-miss would trigger the purchase of additional scratch cards. Within the group exposed to the near-miss, those who purchased more cards appeared to be those who experienced this spike in urge. The purchasers showed far higher urge ratings following the near-miss than the non-purchasers. Furthermore, there was a positive point-biserial correlation between participants’ ratings of their urge to gamble following near-misses and their purchasing behaviour. This lends support to the idea that near-misses trigger increases in the urge to gamble, which can in turn prompt some players to buy more cards.

An unexpected finding concerned those in the loss group. Despite three successive losses in Card 2, eight participants still purchased at least one more card. In the loss group, urge to gamble was significantly lower than in the near-miss group, and (unlike in the near-miss group) there were no differences in the urge ratings between purchasers and non-purchasers. Additionally, urge ratings following losses were uncorrelated with purchasing behaviour. Thus despite not showing an increase in urge to continue gambling, a small subset of people in the loss group *still chose* to purchase additional cards. This puzzling finding hints at the importance of considering other individual differences among players and how these may relate to purchasing behaviours. Some candidate variables that may be informative include impulsivity (MacLaren et al. 2012) and the closely related concept of delay discounting (Dixon et al. 2003; Callan et al. 2011) in which deficits are strongly related to gambling behaviour. Research examining differences in delay discounting have shown that participants who chose to purchase scratch cards from an experimenter in an unrelated experimental context discounted delayed rewards at a steeper rate than those who did not purchase scratch cards (Callan et al. 2011). The inability of some individuals to delay larger, later rewards and instead engage in less-rewarding behaviour in the short term may explain some differences in purchasing behaviour within the current study. Individual differences like delay discounting could also potentially account for why some participants with low urges to gamble nonetheless purchased an extra card (i.e. the purchasers in the loss group), and might also explain why some participants with high urge to gamble following a near-miss might have been able to refrain from making a purchase (they may have been able to discount the slim *possibility* of earning money immediately, for the surety of having an extra $5.00 to spend that evening).

Although not the manipulation of primary interest, the data for Card 1 clearly shows that small wins in scratch card play trigger increases in the urge to gamble—a finding that replicates previous results from our laboratory. For both groups urge was relatively low following the first loss, then rose dramatically following the small win, and dropped once again following another loss. When considering the effects of small wins, it is important to note that the most common outcome in scratch card play is a loss, but if a prize *is* won, the most common prize amount in virtually all scratch card games is in fact not a true win, but rather what in gambling parlance is called a “push”. This outcome is a type of “win” in which the player gains an amount equal to that of their original bet. It is not unreasonable to assume that the majority of these push outcomes may be simply “cashed in” for another card by the player, as they are of equivalent value. Therefore, future research would benefit from designs that directly compare the effects of pushes and *true* wins.

### Limitations

While we tried to accurately approximate real gambling behaviour, it should be reiterated that participants were not gambling with their own money and thus could not truly lose within the constraints of the experimental design. Although our participants all had experience playing scratch cards, it’s possible that different results would be obtained with a community-based sample of more experienced scratch card players, or more experienced gamblers in general. Future studies should attempt to clarify the roles of experience, frequency of play, and gambling status on near-miss effects. Another limitation of the current study is the relatively small jackpot prize that participants could win, in contrast to real scratch card jackpots (which range from tens of thousands to millions of dollars). However, we believe this factor would only serve to attenuate responses to the different outcome types. In this light, the robust effect of near-misses on urge that we obtained may be viewed as a conservative estimate of the effects that may occur in real scratch card games.

## Conclusion

Overall, the results of this study highlight the potentially problematic influence of near-miss outcomes in scratch cards on player behaviour and motivational state. Individuals who experienced near-miss outcomes showed a heightened motivation to gamble. The players who showed the largest urges following the near-miss were those who chose to purchase additional scratch cards. Thus gambling urge appears to be a state related to purchasing behaviour, perhaps lying dormant until triggered by a specific game outcome, such as a near-miss. While some players seek additional gambling opportunities regardless of the outcomes they experience, for others, near-miss outcomes may be just enough to encourage further gambling behaviour through increases in subjective urge related to arousal and frustration.
